# Supplementary material for: SANTA-SIM: simulating viral sequence evolution dynamics under selection and recombination
Source: Virus Evol. 2019 Mar 8;5(1):vez003. doi: 10.1093/ve/vez003 (PMC6407609; doi:10.1093/ve/vez003)
Supplement: Supplementary Data [file vez003_supp.pdf]

SANTA-SIM: Simulating Viral Sequence Evolution Dynamics Under Selection and Recombination: Supplementary material

Abbas Jariani[1,2], Christopher Warth[3], Koen Deforche[4], Pieter Libin[5,6], Alexei J Drummond[7,8], Andrew Rambaut[9], Frederick A Matsen IV[3], and Kristof Theys[5]

[1]Laboratory for Genetics and Genomics, Center of Microbial and Plant Genetics, KU Leuven, 3001 Leuven, Belgium
[2]VIB Laboratory for Systems Biology, 3001 Leuven, Belgium
[3]Program in Computational Biology, Fred Hutchinson Cancer Research Center, Seattle, USA
[4]Emweb, 3020 Herent, Belgium
[5]Laboratory Clinical and Evolutionary Virology, Rega Institute for Medical Research, KU Leuven, Belgium
[6]Artifical Intelligence lab, Department of computer science, Vrije Universiteit Brussel, Brussels, 1000, Belgium
[7]Centre for Computational Evolution, University of Auckland, 1010 Auckland, New Zealand
[8]Department of Biosystems Science and Engineering, Eidgenössische Technische Hochschule, Zurich, 4058 Basel, Switzerland
[9]Institute of Evolutionary Biology, Ashworth Laboratories, University of Edinburgh, Edinburgh EH9 3FL, UK

December 19, 2018

Abstract

1 Benchmarking experiment

We compared performance of SANTA-SIM to three established evolution simulators: simuPOP [PK05], SFS CODE [Her08] and VIRAPOPS [PV13]. We simulated a similar scenario in all four software: evolution over 10,000 generations, in presence of negative selective pressure. A compressed file with the files used for benchmarking accompanies this supplementary file. We carried out such simulation over a range of population sizes and genome lengths. Memory footprint and elapsed time were measured for each simulation. A computer with 3.4 GHz Intel® Core i7 CPU and 32 GB of RAM was used to run the simulations (Ubuntu 16.04, Java openjdk version 1.8).

All simulations are configured to run for 10,000 generations under purifying selection and without sampling the simulated sequences. We did not proceed to fully profile VIRAPOPS as many of the benchmark simulations would take more than a day to run, however, we acknowledge that this might be due to sub-optimal strategy in the choice of simulation configuration. Memory consumption of SANTA-SIM (Figure 1) and SFS CODE (Figure 2) are in similar order of values and scale similarly with increasing sequence length and population size. However, SANTA-SIM in general consumes more memory than SFS CODE which might be because the former is written in Java while the latter is written in C. Memory consumption of simuPOP appears to exceed those of SANTA-SIM and SFS CODE (Figure 3). The running time of SFS CODE was shorter compared to SANTA-SIM, however, SANTA appears to be scaling better for longer sequences. These running times are in the order of minutes, while running times of simuPOP were in the order of hours. Overall, SANTA-SIM has an acceptable performance in comparison to other simulators and scales well with increasing genome lengths and population sizes.

References

[Her08] Ryan Hernandez. A flexible forward simulator for populations subject to selection and demography. *Bioinformatics*, 24(23):2786–2787, December 2008.

[PK05] Bo Peng and Marek Kimmel. simuPOP: a forward-time population genetics simulation environment. *Bioinformatics*, 21(18):3686–3687, 2005.

[PV13] Michel Petitjean and Anne Vanet. VIRAPOPS: a forward simulator dedicated to rapidly evolved viral populations. *Bioinformatics*, page btt724, 2013.

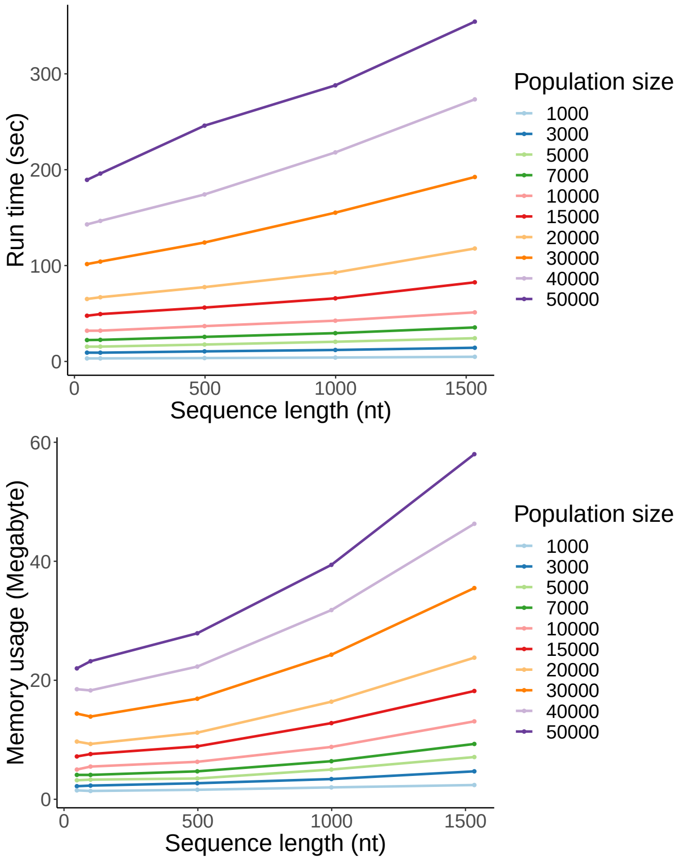

Figure 1: **performance test of SANTA-SIM.**
Memory and run time of simulations with purifying selection for 10000 generations with different population sizes and genome lengths.

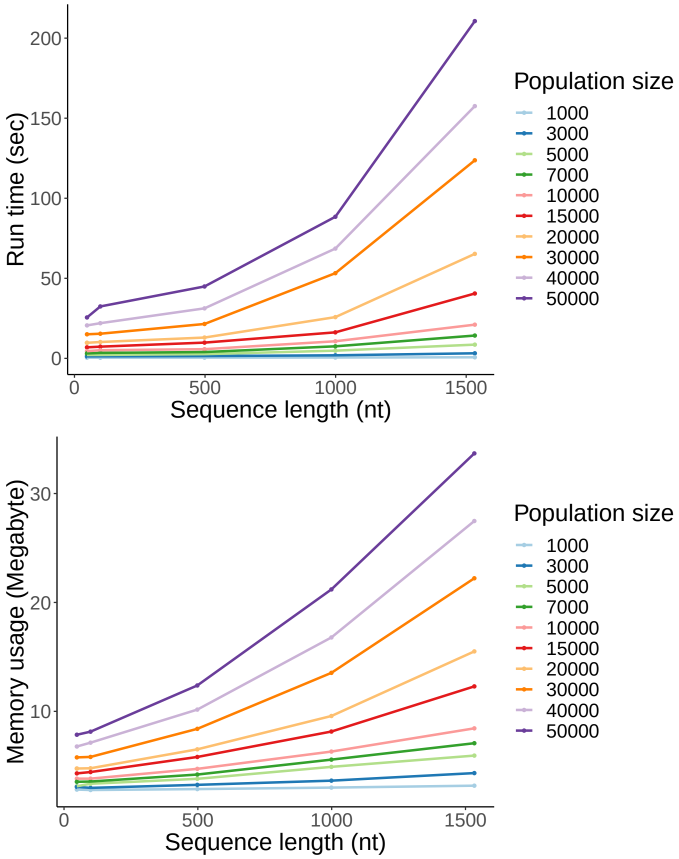

Figure 2: **performance test of SFS CODE.**
Memory and run time of simulations with normally distributed negative selection for 10000 generations with different population sizes and genome lengths.

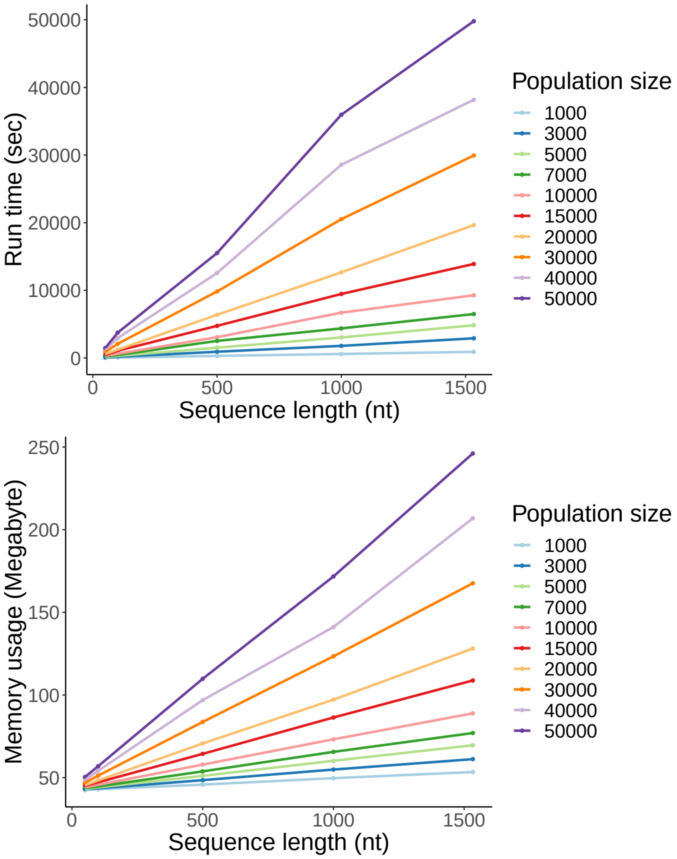

Figure 3: **performance test of simuPOP.**
Memory and run time of simulations with negative selection for 10000 generations with different population sizes and genome lengths. As instructed in the software manual the genome elements are codons with 64 states.
